# *AUREA* maintains the balance between chlorophyll synthesis and adventitious root formation in tomato

**DOI:** 10.1038/s41438-020-00386-x

**Published:** 2020-10-01

**Authors:** Junqing Wu, Jie Cheng, Chunmiao Xu, Shilian Qi, Wenru Sun, Shuang Wu

**Affiliations:** College of Horticulture, FAFU-UCR Joint Center for Horticultural Biology and Metabolomics, Haixia Institute of Science and Technology, Fujian Agriculture and Forestry University, Fuzhou 35002 Fujian, China

**Keywords:** Flooding, Plant signalling, Plant breeding, Plant molecular biology, Gene regulation

## Abstract

Flooding tolerance is an important trait for tomato breeding. In this study, we obtained a recessive mutant exhibiting highly enhanced submergence resistance. Phenotypical analyses showed that this *resistant to flooding* (*rf*) mutant displays slightly chlorotic leaves and spontaneous initiation of adventitious roots (ARs) on stems. The mutation was mapped to the phytochromobilin synthase gene *AUREA* (*AU*), in which a single amino acid substitution from asparagine to tyrosine occurred. In addition to the classic function of *AU* in phytochrome and chlorophyll biogenesis in leaves, we uncovered its novel role in mediating AR formation on stems. We further observed temporal coincidence of the two phenotypes in the *rf* mutant: chlorosis and spontaneous AR formation and revealed that AU functions by maintaining heme homeostasis. Interestingly, our grafting results suggest that heme might play roles in AR initiation via long-distance transport from leaves to stems. Our results present genetic evidence for the involvement of the *AU*–heme oxygenase-1–heme pathway in AR initiation in tomato. As fruit production and yield in the *rf* mutant are minimally impacted, the mutation identified in this study may provide a target for biotechnological renovation of tomato germplasm in future breeding.

## Introduction

Frequent flood occurrences due to global climate change have posed a challenge to crop yield and agricultural productivity^1,2^. Compared to the huge efforts in rice, tomato, a commercially important vegetable, has received little attention with regard to waterlogging resistance^3^. The widespread popularity of tomato around the world has led to its cultivation in a variety of environments. As with many terrestrial plants, most tomato varieties have no flooding resistance and are very sensitive to submergence^4^. Thus, identifying genes that enhance flooding tolerance without affecting productivity is an important strategy to improve tomato adaptability.

Plants have developed different strategies to address submergence. Some crops, including rice, form aerenchyma in root cortex tissues to ensure gas exchange, reducing hypoxic injury. A number of beneficial genes have previously been identified, including *Sub1A*, which mediates the response of plant hormones to lower carbohydrate consumption when plants undergo submergence damage^5^. A more common way in most plants, however, is to enhance the formation of adventitious roots (ARs), which facilitate the absorption of dissolved oxygen and the uptake of minerals in the waterlogging condition^6^.

ARs are generally observed in herbs and woody plants, but the natural occurrence of ARs in dicot vegetables is rare^7,8^. In tomato, waterlogging has been shown to trigger the formation of ARs^6,9^. The mechanism underlying waterlogging-induced ARs often involves multiple endogenous phytohormones^10^. A high level of auxin promotes AR initiation, while an increased ethylene level enhances the sensitivity of plants to the auxin response^11,12^. Other hormones including cytokinin, GA, JA, and ABA all participate in AR formation and often interact with each other during AR development^13–16^. In addition to these phytohormones, a gaseous signaling molecule, carbon monoxide (CO), mainly derived from the decomposition of endogenous heme by heme oxygenase-1 (HO-1) during phytochrome synthesis, has been shown to affect AR formation in incised plant tissues^17,18^. Despite these early physiological works and exogenous treatments, no genetic evidence has been found, and it remains unclear whether endogenous heme or CO functions in vivo to affect AR formation.

In this study, we identified a mutation in the tomato gene *AUREA* (*AU*) that not only led to leaf chlorosis but also caused the spontaneous formation of ARs on stems. Under flooding stress, *au* mutants extensively formed ARs along the stem, while wild-type (WT) plants produced ARs only at the root–shoot junction. After submergence for 7 days, the *resistant to flooding* (*rf*) mutants showed higher sensitivity to flooding regarding AR initiation compared to WT. Interestingly, mutation of the *AU* gene appears to have minimal impact on tomato growth and fruit yield. Further analyses demonstrated that the flooding resistance of *rf* mutants mainly results from the accumulation of heme and enhanced HO-1 activity, both of which function in the phytochrome synthesis pathway. In addition, we show that the exchange of substances, including heme, between leaves and stems accounts for AR formation on stems. Our study provides genetic evidence that the *AU*–HO-1–heme pathway induces ARs in tomato, and our findings offer a target for trait improvement by biotechnology in future tomato breeding.

## Results

### The *rf* mutant is highly resistant to waterlogging

**Fig. 1:**
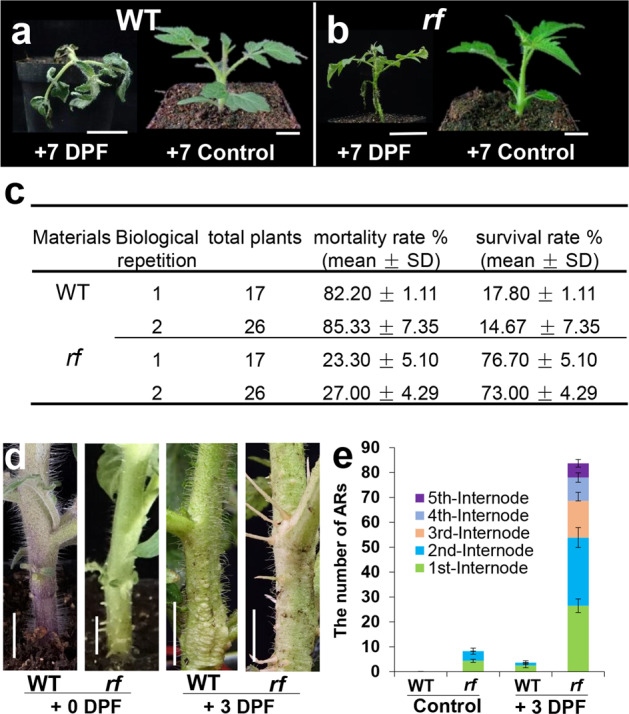
*rf* mutant is flooding resistant. **a**, **b** Flooding resistance of wild-type (WT) and *rf*. Both WT (**a**) and *rf* (**b**) at the three-leaf stage were treated by flooding for 7 days. Seedlings of WT (**a**) and *rf* (**b**) at the same stage without the flooding treatment were used as the control. Bars = 1 cm. **c** Quantification of survival and mortality rate at 7 days after flooding treatment. The results are based on two independent treatments (*n* = 17 and 26, respectively. Mean ± SD). **d** AR formation in the hypocotyl of WT and *rf* exposed to 0 or 3 days of flooding treatment. Bar = 1 cm. **e** Quantification of adventitious roots in WT and *rf* exposed to 0 or 3 days of flooding treatment. Error bars represent SD (*n* = 15). AR adventitious root, DPF days post flooding

**Fig. 2:**
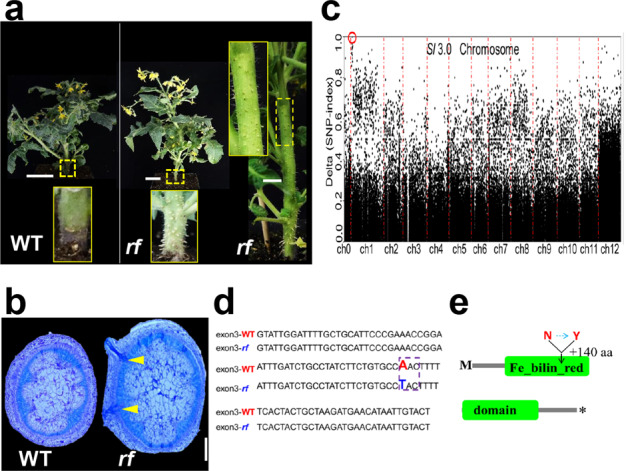
Phenotypic analysis and map-based cloning of the candidate gene. **a** Flowering plants of WT and *rf* grown under normal conditions. The yellow dotted boxes indicate the hypocotyl or distal stem internodes that enlarged in the corresponding insets. Bar = 5 cm. **b** Histological sections of hypocotyl ARs in WT and *rf*. The yellow arrows point to ARs initiated in the pericycle-like layer. Bar = 1 mm. **c** SNP-index peak map of the mutation in *rf* by BSA-seq. The red circle indicates the collection of points with a 100% SNP index. **d** Location of the single-nucleotide mutation in the third exon of the candidate gene. The dotted box indicates the mutated codon. **e** Schematic structure of the AU protein and the position of the single amino acid substitution in the Fe-bilin-red domain

To evaluate the flooding effect on tomato growth, we treated WT tomato plants with water flooding stress and observed growth on different days. At 5 days post flooding (DPF), the tomato plants became wilted, and at 7 DPF, most plants died (Fig. 1a, c). Using the same flooding conditions, we screened a tomato EMS library^19^ and isolated a mutant that exhibited high flooding tolerance. We named the mutant *rf*. At 7 DPF, the *rf* mutant remained alive with only slight wilting (Fig. 1b), with a survival rate greater than 73% (Fig. 1c). In contrast, WT generally died after 7 DPF, with an over 80% mortality rate (Fig. 1c). Interestingly, *rf* also exhibited two other phenotypes: chlorosis (Fig. 1b, right) and spontaneous formation of ARs (Fig. 1d, left). Under normal conditions, no AR was detected on the stem of WT tomato, and at 3 DPF, only a few ARs on WT hypocotyls were induced (Fig. 1d). In contrast, under the same growth conditions, *rf* formed a large amount of ARs on hypocotyls and stems above the cotyledons (Fig. 1d). We further quantitatively compared the number of ARs between WT and *rf*. Almost no AR formed on the first two internodes of flowering WT (Fig. 1e), but *rf* formed ARs as early as the three-leaf stage and had developed approximately eight ARs at the flowering stage (Fig. 1e). AR formation in *rf* mutants was also visible in more expanded regions of the most distal stem internode (Fig. 2a, right). Our observations showed that the *rf* mutation not only increased the amount of flooding-induced ARs but also substantially expanded the region of AR formation along the stem: a large number of ARs (approximately six) were visible even on the fifth distal internode (Fig. 1e).

To further determine whether chlorosis in *rf* also contributes to flooding resistance, we artificially created etiolated seedlings by growing WT tomato in darkness and then treated them with flooding. As shown in Supplementary Fig. S1, yellow tomato seedlings (Y-WT) were even more susceptible than were normal WT seedlings to flooding stress, and at 3 DPF, most Y-WT seedlings were almost dead. It appears that the etiolation reduced the resistance of tomato plants to waterlogging. Thus, we infer that the producing ARs may confer resistance to submergence in *rf* mutants.

### *AU* is the candidate gene of *rf*

To identify the causative mutation that accounts for the AR phenotype, we developed an F_2_ mapping population by crossing the *rf* mutant with WT. F_1_ plants from the crossing exhibited the normal phenotype, and the segregation ratio of AR to non-AR plants in the F_2_ population conformed to Mendel’s separation law (3:1) based on the *χ*^2^ test (*χ*^2^_229_ = 0.118, *χ*^2^_0.05,2_ = 3.84) (Fig. 2a, b; Supplementary Fig. S2a). Thus, the AR phenotype in *rf* is the result of a recessive allele. Using next-generation sequencing-based bulked-segregant analysis (BSA), we identified a linkage peak on the short arm of chromosome 01 (Fig. 2c). We then analyzed the SNP index of all SNP loci and identified two with a 100% SNP index (Supplementary Fig. S2b). The functional annotation indicated that only one SNP is located in the gene coding sequence (Supplementary Fig. S2c), and our further analysis assigned the SNP to the third exon of gene Solyc01 g008930 (Fig. 2d). The mutation results in an amino acid substitution from asparagine (N) to tyrosine (Y) (Fig. 2e). According to the tomato reference genome sequence (https://www.solgenomics.net/), Solyc01 g008930 encodes a phytochromobilin (PΦB) synthase gene *AU*, which is homologous to *AtHY2*. The SNP is located in the Fe-bilin-red domain of the AU protein (Fig. 2e). Previous studies have revealed the association of the chlorosis phenotype with the *AU* mutation^20^. However, the biological function of *AU* in mediating AR occurrence has not been reported.

### Functional complementation in the *rf* mutant

**Fig. 3:**
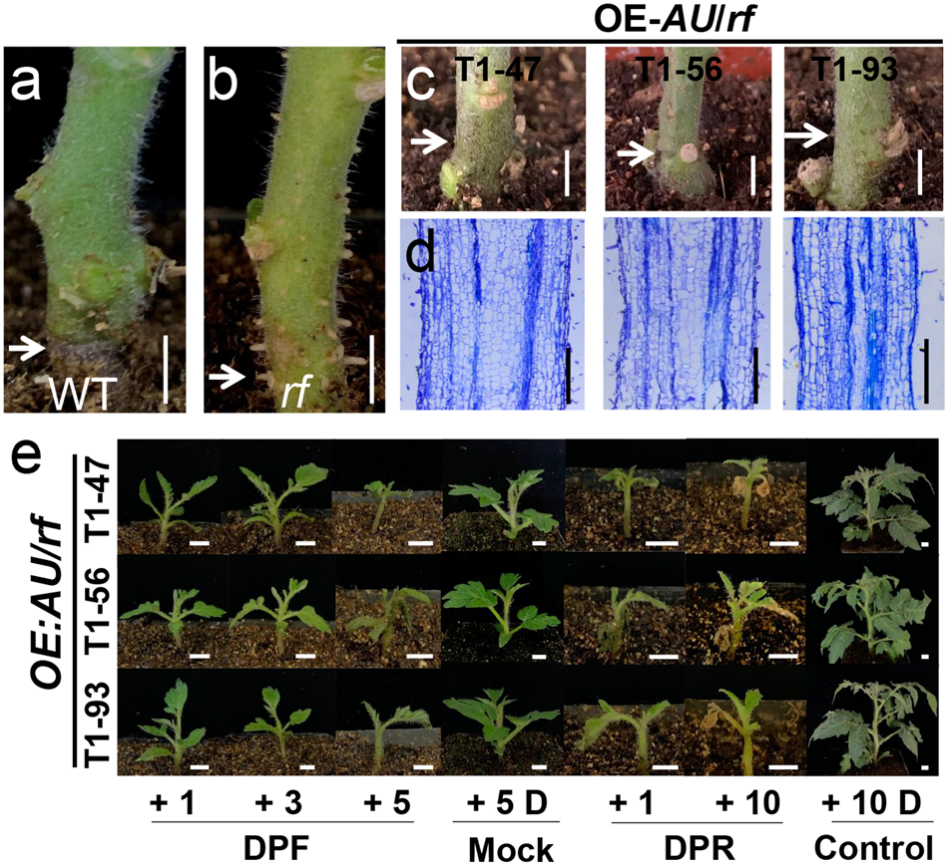
Genetic complementation of *rf*. **a**, **b** The AR phenotype in WT and *rf*. Hypocotyl-derived ARs (**c**) and histological sections of AR primordia in *OE-AU/rf* (**d**). T1-47, 56, and 93 represent three T1 lines from different independent T0 plants. **e** Test of flooding resistance in *OE-AU/rf* lines at 1, 3, and 5 DPF and 1 and 10 DPR (days post recovery). Seedlings were treated with 5 days of flooding and then transferred to normal conditions for recovery. Bar = 1 cm

To functionally verify that *AU* participates in AR formation, we cloned the full-length cDNA of *AU* and expressed it under the 35S promoter in the *rf* mutant (*OE-AU/rf*). We obtained seven independent stably transgenic tomato lines with *OE-AU* expression in the *rf* mutant background (*OE-AU/rf*). We verified that all these lines significantly overexpressed the gene (increased by 3–700-fold). Different from the case in WT (Fig. 3a) and *rf* (Fig. 3b), we could not detect any AR emergence in these complemented lines. We also verified this complementation in the next T_1_ generation, as shown in Fig. 3c. To determine whether the absence of ARs is due to blocked AR emergence or initiation, we examined the anatomic section of three *OE-AU/rf* lines (T1-47, 56, 93) (Supplementary Fig. S3). In the hypocotyls of all these lines, we failed to detect any AR primordia (Fig. 3d), suggesting that AR imitation was entirely repressed.

In addition, we found no significant difference from WT in the chlorophyll content of all *OE-AU/rf* lines (Supplementary Fig. S4a). However, surprisingly, the *OE-AU/rf* lines exhibited a higher survival rate than WT after 5 DPF (Fig. 3e). One possibility is that the diameter of the first four internodes of all transgenic lines was considerably larger than that in WT (Supplementary Fig. S5a). This expanded radial structure might confer higher resistance under flooding stress. The other possible contributing factor might be the increased leaf area in *OE-AU/rf* lines (Supplementary Fig. S5b). We next compared how fast different tomato plants recover from submergence stress. We treated both *OE-AU/rf* and *rf* plants with flooding for 5 days and transferred them back to normal conditions to assess their recovery rate. The *rf* mutant regained vitality as early as 3 day post recovery (DPR) and reached an approximately normal status after 7 DPR (Supplementary Fig. S6b); however, the growth of all *OE-AU/rf* lines was still seriously impaired even at 10 DPR (Fig. 3e). WT plants were mostly dead after 5 DPF (Supplementary Fig. S6b) and only survived until 3 DPF (Supplementary Fig. S6a). Therefore, *rf* was more resistant than both WT and the overexpression lines, which supported the hypothesis that the null *au* allele was essential for AR-dependent flooding resistance. Therefore, our results suggest that *AU* plays dual roles in promoting chlorophyll synthesis in leaves and repressing AR formation in stems.

### Heme accumulation possibly accounts for AR initiation

**Fig. 4:**
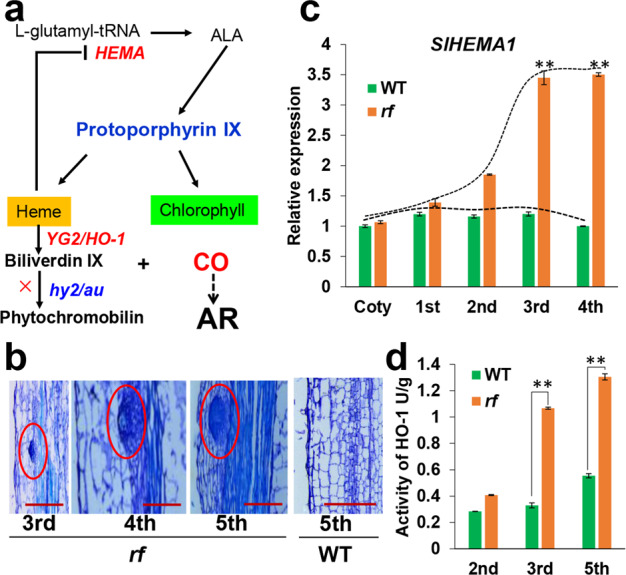
Dual roles of *AU* in chlorophyll synthesis and AR primordia initiation. **a** Schematic diagrams of *AU* function in the heme pathway in tetrapyrrole metabolism. **b** Histological analysis of AR primordia in the hypocotyl of *rf* at the three- to five-leaf stage. Five-leaf-stage WT plants were used as controls. The red circle shows the AR primordium, and the number below each figure represents the corresponding leaf stage. Bar = 0.2 mm. **c** The temporal expression pattern of *SlHEMA1* in WT and *rf*. The number below each column represents the corresponding leaf stage. Error bars represent SE (*n* > 3). **d** Comparison of HO-1 activity between WT and *rf* at the two- to four-leaf stage. The number below each column represents the corresponding leaf stage. Error bars represent SE (*n* > 3)

Chlorosis is presumably derived from chlorophyll deficiency in leaves, while the extra ARs in *rf* mutants are formed in the pericycle-like cells of the stem (Fig. 2b)^21,22^. Despite developing in distinct tissues, ARs and chlorosis could still be associated since both are controlled by the same gene. *AU* functions in the heme metabolic pathway for biliverdin IX decomposition; thus, we inferred that the loss of function of *AU* might block phytochrome synthesis and possibly lead to the accumulation of heme (Fig. 4a), one of the upstream intermediates of *AU*. Interestingly, a previous report showed that exogenous heme increased the number of ARs in the incision of plant explants^17^. The extra heme might suppress the expression of *HEMA*^23^, a key enzyme catalyzing the upstream substrates of the tetrapyrrole metabolic pathway, and lead to a feedback loop in the tetrapyrrole pathway (Fig. 4a). Thus, the accumulation of heme due to the disrupted *AU* may not only induce AR initiation but also decrease the metabolic efficiency of the entire tetrapyrrole pathway, ultimately reducing the chlorophyll content.

To compare the time course of extra AR formation and chlorophyll change, we first produced paraffin sections of *rf* mutants at different developmental stages. The AR primordium was initially detected at the three-leaf stage and became easily visible at the five-leaf stage (Fig. 4b). Initial AR emergence was rare, with an average of 1 AR per plant at the three-leaf stage, but they developed quickly, with over seven ARs per plant at the five-leaf stage (Supplementary Fig. S4b). Consistently, the change in chlorophyll content appeared to have a similar trend, with a gradual increase from the two- to five-leaf stage (Supplementary Fig. S4a). In line with the chlorophyll change, we also detected a gradual rise in *SlHEMA1* expression from the two- to four-leaf stage (Fig. 4c), and the activity of HO-1 also increased after the two-leaf stage (Fig. 4d). These results suggest a heme-mediated correlation between ARs and the chlorosis phenotype of *rf* mutants. We inferred that the additional heme derived from the *AU* mutation might decrease chlorophyll synthesis by repressing *SlHEMA1* expression but is then decomposed by HO-1. As a result, AR primordium initiation is mostly synchronized with the increase in chlorophyll.

### HO-1 activity positively impacts AR abundance

**Fig. 5:**
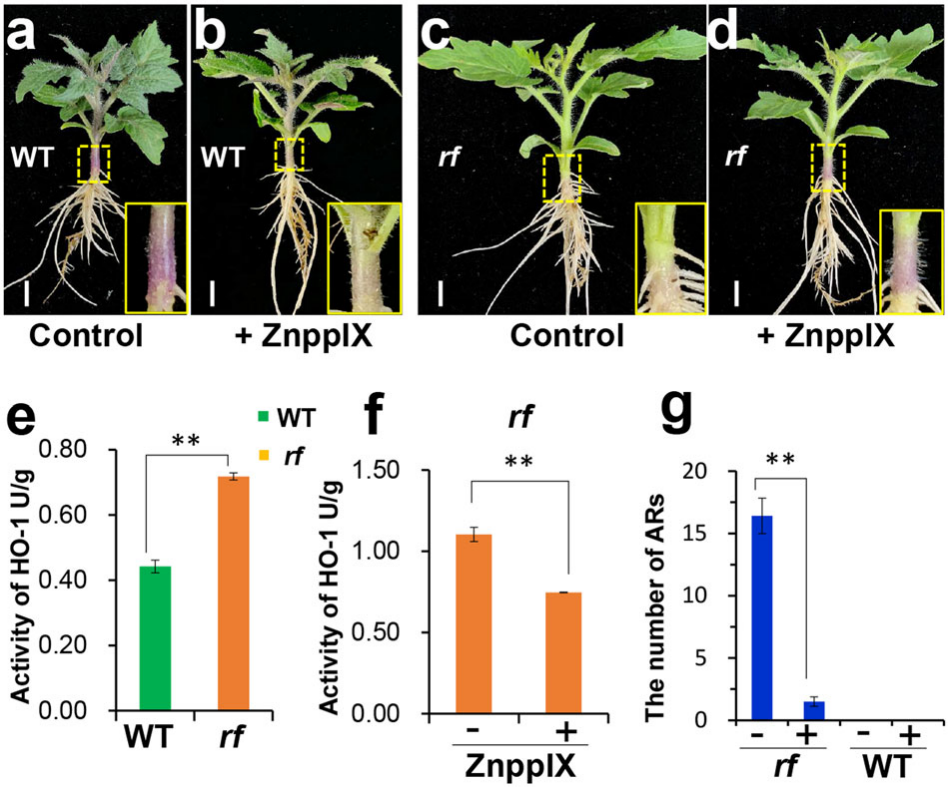
HO-1 activity positively regulates AR formation. Hypocotyl-derived ARs in WT (**a**, **b**) and *rf* (**c**, **d**) treated with 200 µM ZnPP IX for 5 days. ZnPP IX is an inhibitor of HO-1 activity. Bar = 1 cm. **e** HO-1 activity increases in the *rf* hypocotyl compared to the WT under normal growth conditions. Error bars represent SE (*n* = 3). **f** ZnPP IX decreases HO-1 activity in the *rf* hypocotyl. Error bars represent SE (*n* = 3). **g** The numbers of AR in WT and *rf* hypocotyls with (+) or without (−) ZnPP IX treatment. Error bars represent SE (*n* = 3). ZnPP IX zinc protoporphyrin IX

As catabolism of heme by HO-1 is necessary for the biological function of heme in plants, we examined HO-1 activity in the hypocotyls and detected higher HO-1 activity in *rf* than in WT (Fig. 5e). To further verify the involvement of HO-1 in heme-induced AR formation, we treated both WT and *rf* with ZnPP IX (Fig. 5a–d), an inhibitor of HO-1 activity. The application of ZnPP IX remarkably suppressed the number of ARs in *rf* (Fig. 5c, d and g), correlating positively with decreased HO-1 activity (Fig. 5f). These results provide evidence supporting that HO-1 activity promotes AR occurrence in *rf*.

### Communication between leaves and stems activates AR initiation

**Fig. 6:**
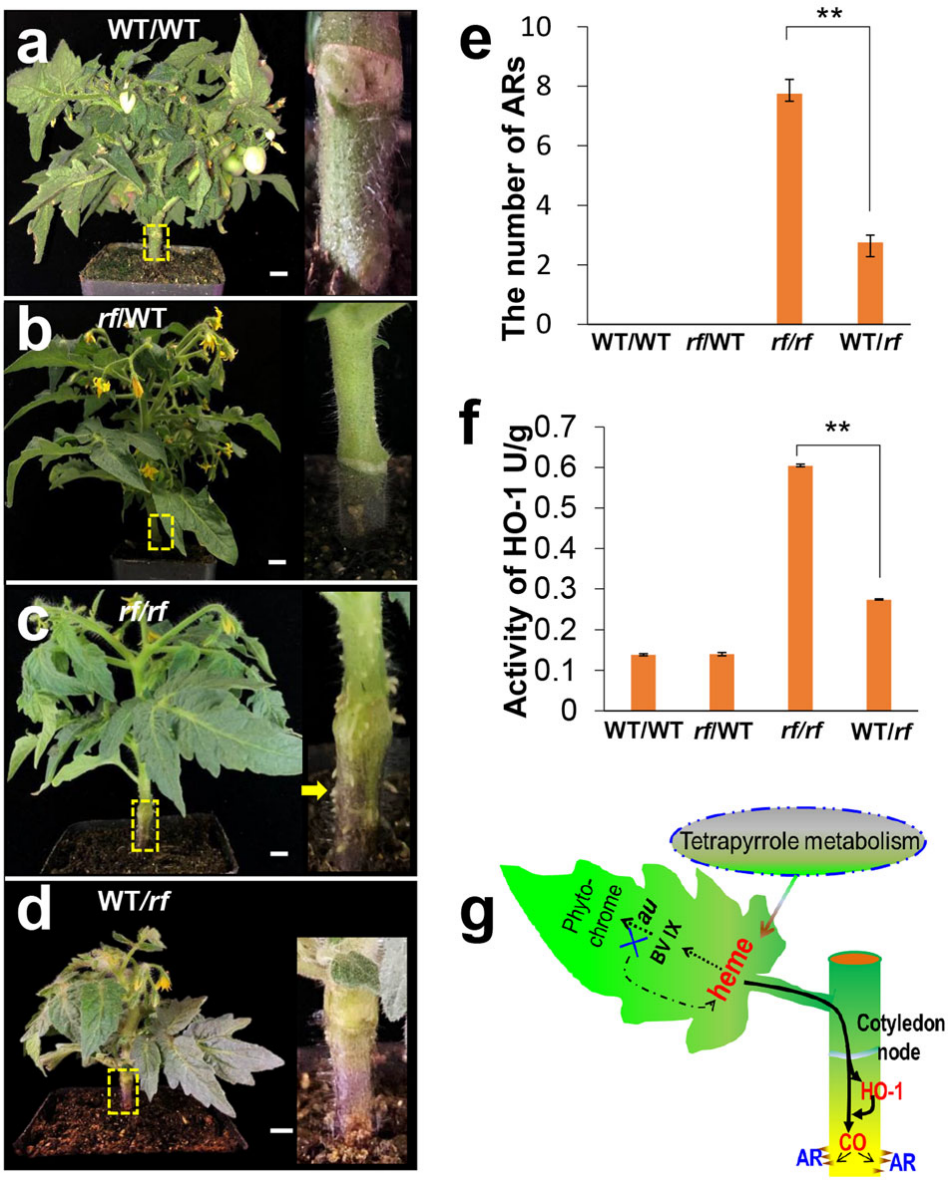
Communication between leaves and hypocotyls is responsible for AR initiation. **a**–**d** Different types of grafting combinations. *rf*/WT represents that scion of *rf* was grafted onto the WT stock, and the same designation rule applies to WT/WT, WT/*rf*, and *rf*/*rf*. The yellow boxes indicate the hypocotyl section that is enlarged in the corresponding insets. The yellow arrows indicate adventitious roots (**c**). Bar = 1 cm. **e** Quantification of ARs in different types of grafting. Error bars represent SE (*n* = 5). **f** Hypocotyl HO-1 activity in different types of grafting. Error bars represent SE (*n* = 5). **g** Schematic diagram of the *AU*–(HO-1)–heme–CO pathway in adventitious root formation. Loss of function of *AU* potentially leads to massive accumulation of heme in leaves and enhances HO-1 activity in the hypocotyl. The overaccumulated heme fluxes from the leaves to hypocotyls, where it is decomposed by HO-1. Through CO production, extra heme promotes the formation of adventitious roots in stems

In the metabolic network, derivatives of the *AU* mutation, such as heme, mostly accumulate in leaves because tetrapyrrole metabolism mainly occurs in the plastids of green leaves, providing the primary substrates for phytochrome and chlorophyll synthesis. However, ARs are initiated in the pericycle-like cells of *rf* stems (Fig. 2b), where few photosynthetic cells develop to produce sufficient derivatives to induce AR initiation. Therefore, we sought to determine whether there is crosstalk or metabolite exchange between leaves and stems, which might contribute to AR induction. To this end, we applied the grafting approach to test this hypothesis. To rule out wounding-induced ARs, we examined the WT hypocotyl after grafting and did not detect any ARs on hypocotyls when WT was grafted onto WT (Fig. 6a). However, surprisingly, the scion of *rf* also failed to stimulate the ARs on the hypocotyl of stock WT (Fig. 6b). We then grafted the scions of WT and *rf* to *rf* stock and found that the hypocotyl of *rf* stocks produced ARs regardless of the type of scions grafted (Fig. 6c, d). Interestingly, compared with the hypocotyl ARs of normal *rf* plants (approximately five ARs) (Fig. 1e), grafting with the scion of WT had decreased AR numbers on *rf* hypocotyls (approximately three weak ARs), while the scion of *rf* had increased AR numbers (approximately eight ARs in) on the hypocotyl of *rf* stocks (Fig. 6e). Although this result provides preliminary evidence supporting the existence of crosstalk between leaves and hypocotyls, it remains unclear why *rf* scion failed to induce ARs on WT stocks. We then further quantified the activity of HO-1 among different types of grafting. Consistent with the results of ARs, there was no difference in HO-1 activity between the stocks of WT with different types of scions grafted (Fig. 6f). However, we detected strong HO-1 activity in the *rf* stocks grafted with the scions of WT and *rf* (Fig. 6f). The HO-1 activity in the *rf* stocks seemed to be repressed when grafted with WT scions, which was also consistent with the change in ARs in the grafting experiments (Fig. 6e). Thus, we inferred that crosstalk exists between leaves and hypocotyls and that it is necessary for AR initiation on stems. Furthermore, active HO-1 is necessary for the scion of *rf* to stimulate ARs in the stock of WT, but there is insufficient HO-1 in the WT hypocotyls to initiate AR primordium.

## Discussion

### Novel roles of *AU* in AR development

AR formation is a complex process and is controlled by multiple environmental and endogenous factors, such as light, phytohormones, temperature, sugar, and other small molecules^14,24–27^. Auxin is one of the most important phytohormones that promotes ARs, and many previous studies on de novo root regeneration have indicated that different early signals need to be converted into the auxin response during AR formation^28,29^. In cucumber cuttings, exogenous NPA, an auxin transport inhibitor, not only repressed the auxin dependent AR numbers but also decreased the endogenous HO-1 activity as well as CO content. In addition, exogenous NO and CO solutions were both shown to increase the AR numbers^17,30^. Therefore, both HO-1 activity and CO content can have positive roles in the process of AR initiation.

In both animals and plants, HO is regarded as the primary source for endogenous CO production, which is an intermediate metabolite in the Fe branch of the tetrapyrrole metabolic pathway^31^ and is derived from the decomposition of heme by HO-1. In this study, we demonstrated that the activity of HO-1 is positively associated with AR formation, consistent with a previous report that exogenous hematin induces HO-1 activity and increases AR numbers in cuttings^32^. Based on the change in HO activity in *rf*, the fact that CO is the major product of heme/HO, and the evidence that CO acts as the signaling molecule to induce AR formation, we speculate that CO is likely functional in the heme/HO pathway to induce AR initiation in *rf* mutants.

Previously, it was also shown that HO-1 positively regulates lateral root (LR) initiation^33,34^. However, no genetic evidence has been presented in planta that the accumulation of endogenous heme and the activity of HO-1 affect AR induction. Here, we demonstrate that *AU* not only functions in leaf chlorosis but also in AR development in stems. Interestingly, the *OE-AU/rf* lines showed the same effect on stem diameter and leaf area as the *rf* mutants, both of which were significantly larger than that of WT (Supplementary Fig. S5b). This apparent paradox might derive from the potential link between phytochrome photoreceptors and leaf areas, which has been previously reported in *Arabidopsis*. For example, leaf area in *phyB* mutants is greatly reduced^35^, and overexpression of phytochrome C increases the primary leaf area^36^. It is well known that *AU* helps to improve phytochrome by generating the direct precursor phycocyanobilin. Thus, overexpression of *AU* might augment the aerial biomass (exemplified by the enlarged leaf area and stem diameter) by increasing the level of phytochrome photoreceptors.

### AR formation is uncoupled from the chlorosis phenotype

Although the connection between disrupted *AU* function and leaf chlorosis has been previously reported^20,23^, AU activity in AR initiation has not yet been discovered. The chlorophyll content of *rf* was lower than that of WT before the three-leaf stage, when ARs were not visible. However, after this stage, the chlorophyll content in *rf* started to increase, which was coincident with the occurrence of ARs. This phenomenon suggests a correlation between chlorosis and AR initiation. Nonetheless, chlorosis is not directly involved in AR formation and submergence resistance. Etiolated tomato seedlings formed no ARs on their hypocotyls, and these seedlings even exhibited weaker *rf*. We thus inferred that the extra ARs induced by the *AU* mutation might be the major factor that conferred submergence adaption.

Downstream of tetrapyrrole metabolism are two different pathways (Mg branch and Fe branch) that share a common substrate, protoporphyrin IX^37^. Accordingly, any factor influencing the efficiency of tetrapyrrole metabolism would also affect phytochrome and chlorophyll synthesis. It has been known that the *au* mutation might induce a feedback response to repress the metabolic conversion from ALA to protoporphyrin IX in the tetrapyrrole pathway^38^. In addition, phytochrome is necessary to activate ALA synthesis^23^, and heme accumulation inhibits glutamyl-tRNA reductase activity^39,40^. Therefore, we conclude that the decreased chlorophyll content at the early stage was mostly derived from the repression of tetrapyrrole metabolism, which presumably resulted from *AU* mutation-induced heme accumulation and phytochrome deficiency. However, the high heme level enhanced HO-1 activity, which helped to release tetrapyrrole repression and promoted AR formation. This may be the reason that accelerated AR formation occurred along with the recovery of chlorosis in *rf* mutants. Together, we think that the ARs produced by *rf* mainly resulted from the defective Fe branch of tetrapyrrole metabolism by the null mutation of *AU*, which might affect chlorophyll synthesis of the Mg branch via a feedback effect on tetrapyrrole metabolism.

### *AU*–HO-1–heme–CO is a possible in planta pathway promoting ARs

*AU* functions downstream of HO-1 in phytochrome biosynthesis^41^. HO-1 decomposes heme to biliverdin IX for AU catalyzed PΦB synthesis, and PΦB is the direct substrate for phytochrome synthesis in plants^42^. Thus, both AU and HO-1 are essential for controlling the types and quantities of phytochromes in plants. Phytochrome synthesis catalyzed by AU and HO-1 mainly occurs in the plastids of the green leaf, while AR formation is restricted to stems. This spatiotemporally distinct location prompted us to hypothesize that the upstream intermediates of AU, such as heme and CO, may flow from leaves to hypocotyls or stems, which is necessary for HO-1 activation and AR formation. This is supported by the result that HO-1 activity was enhanced in *rf* hypocotyls (Fig. 5e). It was also previously verified that HO-1 is involved in LR initiation under ROS induction^43^. It is likely that the additional heme flowing into hypocotyls was decomposed by active HO-1, which is responsible for AR initiation. One of the downstream products of HO-1 activity is CO, a gas signal involved in many aspects of plant development^44,45^. It was previously discovered that exogenous CO solution is able to release the repression of ZnPP IX in AR formation in cuttings^17^.

Fe is one of the byproducts of HO-1-catalyzed heme decomposition. The application of exogenous Fe has been previously reported to stimulate AR initiation in leafy cuttings of Petunia hybrids^27^. It is also possible that local Fe accumulation is involved in AR induction in *rf* mutants. Regardless, several results argue against this possibility. We usually grew all plants in a complete nutrition solution, including for the flooding treatment, which helped to avoid the interference of potential nutrient deficiency. By these treatments, we showed that WT was not flooding tolerant, indicating adequate mineral nutrients (including Fe, NH4+, etc.) were unable to support AR formation through the active absorption of Fe. In contrast, small molecules such as CO that are not absorbed from the environment can trigger AR formation. Thus, we inferred that CO rather than Fe is more likely to participate in AR initiation. In addition, Fe has less mobility in plants, and due to its readily diffusible property, CO is more likely to function in inducing AR in plants.

Here, we provide genetic evidence showing that mutation of *AU* possibly leads to heme accumulation and enhanced HO-1 activity in hypocotyls, stimulating AR initiation. Taken together, our results revealed a potential in planta pathway consisting of *AU*–(HO-1)–heme–CO in AR development.

### Communication between leaves and stems is essential for AR initiation

Since the initial site of heme accumulation is presumably leaves, long-distance transport of heme appears to be necessary for AR formation on hypocotyls. Grafting is a technique that has been widely used to ameliorate the poor traits of cultivated varieties in agriculture and to improve communication between different genetic materials^46,47^. In this study, we conducted grafting between *rf* and WT to confirm substance exchange between leaves and hypocotyls in *rf*, which had a positive effect on AR induction. Although our results support that the heme from *rf* leaves is necessary for activating HO-1 and AR initiation in stems, it remains unclear why *rf* scions failed to stimulate HO-1 activity and AR primordia on WT stocks. It is likely that the heme from *rf* leaves alone is essential but insufficient to induce AR initiation in hypocotyls. The different HO-1 activity between *rf*/WT and *rf*/*rf* reinforces that genetic background also determines HO-1 activity in hypocotyls.

As plants are sessile organisms, they have evolved complex mechanisms to facilitate adaptation to environments. Both the primary root and LR are mainly responsible for absorbing and anchoring^48^. One significant role of AR is to enhance plant resistance under flooding stress. Although spontaneous ARs can occur in the hypocotyl of *Arabidopsis* and many factors have been shown to affect wound-induced ARs^49^, it has rarely been reported that leaf-derived chemicals act in a long-distance manner to influence AR formation on hypocotyls. It is noteworthy that the contribution of leaf-derived auxin and sugars to AR formation in the stems of cuttings has also been shown^50–52^. In this study, we highlight the importance of other metabolic substances by showing that accumulation of heme metabolic intermediates in leaves can activate HO-1 activity in hypocotyls. Indeed, grafting between *rf* and WT confirmed that the potential communication between leaves and hypocotyls plays an important role in AR formation. Thus, our results provide additional evidence suggesting that metabolites derived from leaves can affect the development of stems, such as the formation of ARs.

### *AU* can be a target for biotechnological renovation in tomato breeding

In the past, there has been rapid implication of biotechnology in improving agricultural traits. An optimal target for biotechnologically renovated traits should not affect the normal growth of crops. Our results indicate that *AU* mutation does not reduce the weight per fruit (Supplementary Fig. S7a) or decrease the total number of fruits per plant (Supplementary Fig. S7d), with a WT-like fruit yield per plant in *rf*. Although the chlorophyll content declined in *au* mutants before the five-leaf stage (Supplementary Fig. S4a), the overall fruit production seemed to be unaffected. One possible reason is that the increased stem diameter and leaf area in *rf* mutants might contribute to complementation of the possibly reduced photosynthesis. In addition, the fresh shape index of *rf* was greater than 1, while that in WT was less than 1 (Supplementary Fig. S7c). Thus, the traits caused by *AU* mutation include stronger flooding resistance, WT-like fruit production, and a high fresh shape index and plant height (Supplementary Fig. S7b). Therefore, *rf* could be used as an excellent germplasm in tomato breeding. Moreover, the recessive nature of the AU mutation with a single amino acid change makes it a good target for advanced biotechnology-aided tomato breeding.

Furthermore, we showed the efficiency of grafting in this study. Although the WT scion decreased AR number in the *rf* stock, the actual AR number after grafting was still more than that of the WT grown under the same conditions. Thus, *rf* also provides a quick method to improve the submergence resistance of different tomato varieties by *rf* stock grafting.

## Conclusion

Our study reveals two links that have not been clearly addressed before: the connection between phytochrome metabolism and AR formation and the communication between leaves and stems promoting ARs. In this study, we deduced that the loss of function of *AU* possibly led to the massive accumulation of heme in leaves, which was transported long distances from leaves to stems, where the high heme level enhanced HO-1 activity (Fig. 6g). As a result, the excess heme in stems was decomposed by high HO-1 activity, and the emission of CO, one of the downstream byproducts of heme decomposition, induced the initiation of ARs in stems^17,18^. Since ARs abundantly occurred in *rf* mutants after the three-leaf stage, we speculate that the CO effect on AR initiation is dosage dependent. Due to the relatively small size of younger seedlings (before the three-leaf stage), they possibly had insufficient heme fluxed into stems for AR stimulation. Our findings provide potential genetic evidence supporting the possibility of the endogenous *AU*–HO-1–heme–CO pathway in AR induction in tomato (Fig. 6g). As *rf* mutants had no detectable defects in overall growth and fruit yield, the mutation in *rf* provides an excellent target for future biotechnological tomato breeding.

## Materials and methods

### Plant materials and growth condition

Tomato (*Solanum lycopersicum*) WT and mutant *rf* (TOMJPW1898-1) seeds were obtained from TOMATOMA (http://tomatoma.nbrp.jp/), and *rf* was isolated by screening an EMS library generated in the cultivar Micro-Tom (TOMJPF00001)^19^. All materials were grown in a greenhouse at 28 °C with 16 h in light and 18 °C with 8 h in darkness. All plants were planted in 9 × 9 × 9-cm pots with a standard substrate (PINDSTRUP). The seedlings were grown with Hoagland nutrient solution with light intensity at 5000 Lx.

### Construction of mapping population and BSA

To map the candidate gene responsible for *rf*, the mutant was crossed with WT, and the F_1_ plants were self-fertilized to generate the F_2_ population. The segregation ratio of F_2_ was analyzed by the chi-square test. Genomic DNA was extracted from 55 individuals with mutant phenotypes in the F_2_ population and 55 WT individuals by a modified CTAB method.

The qualified DNA was mixed to construct mutant-like bulk and WT bulk. Both the sequencing parameters and analysis methods were based on Chang et al.’s study^53^. The ORF with an ~100% SNP index in the mutant-like bulk was used for mutation identification and complementary verification.

### Plasmid construction and transformation

Gateway^®^ technology was used for cloning for complementation experiments of candidate genes in *rf*. The full-length cDNA of *AU* was first cloned into the entry vector pDONR221 (the primers are listed in Supplementary Table S2). *AU* was then gateway-cloned into the expression vector pHellsgate 8 driven by the CaMV 35S promoter. The tomato *rf* mutant was transformed using *Agrobacterium tumefaciens* strain C58.

### Treatments and analysis

To obtain tomato lines with waterlogging resistance, tomato plants from the EMS library and WT at the stage of early flowering were treated with 0 and 3 days of flooding. The flooding treatment was applied in plastic trays (80 × 60 × 40 cm) with water deep enough to completely submerge the whole plants, with the water surface less than 1 cm above the plant top. Growth conditions were evaluated, and the total number of ARs on each plant was counted. At least seven plants for each variety were evaluated. For further flooding resistance verification, both WT and *rf* were treated with flooding for 0, 1, 3, 5, and 7 days. Plants exposed to 3 and 5 days of flooding were used for the recovery experiment under normal conditions. Plant viability was evaluated to confirm the resistance capability of different tomato lines.

Drug treatment was conducted at the five-leaf stage. WT and *rf* were grown in half-strength Hoagland nutrient solution and treated with 200 µM zinc protoporphyrin IX (ZnPP IX, Santa Cruz, sc-200329, USA) for 5 days. The ZnPP IX powder was first dissolved in DMSO to prepare a 5 M stock solution. We then added a certain amount of the stock solution directly to the hydroponic nutrient solution for a final concentration 200 μM. The number of ARs was quantified, and the middle section of hypocotyls (~5 mm length) where the ARs occurred was collected for the measurement of HO-1 activity.

### HO-1 activity analysis

We chose five tomato plants with similar growth conditions and collected their hypocotyls for the measurement of HO-1 activity. In all these experiments, we chose the hypocotyl of the rootstock for the measurement. HO-1 activity was assayed and calculated according to a previous method^54^. The reaction was carried out at 37 °C for 30 min after NADPH was added to the enzyme extract. The final production of biliverdin IX was used to evaluate the activity of HO-1. Fresh hypocotyl tissue (1 g) was quickly ground into powder in liquid nitrogen, extracted immediately with 30 mL ice-cold isolation medium, and then filtered into a centrifuge tube through four layers of filter cloth. The samples were centrifuged at 1300 × *g* for 30 min. The supernatant was transferred to a new centrifuge tube followed by centrifugation at 60,000 × *g* for another 30 min, and the final supernatant was used for HO activity measurements.

### Paraffin sectioning

Hypocotyl sections were fixed in FAA (50% ethanol, 5% glacial acetic acid, and 3.7% formaldehyde mixture) overnight at 4 °C. After dehydration in different concentrations of alcohol (15, 30, 50, 75, and 100%), the sample was embedded in wax. A Leica EG1150 was used to cut 10-μm-thick sections, which were stained with 0.5% toluidine blue until the tissue outline was clearly visible. All stained sections were used in hypocotyl AR primordium detection under a stereomicroscope (Leica M205 FA).

### Measurement of chlorophyll

We selected 12 tomato plants with very similar growth conditions and mixed their leaves for chlorophyll measurement. Leaves (0.1 g) from WT, *rf*, and *OE-AU/rf* lines were collected with major veins removed. Chlorophyll was extracted with 10 mL 96% alcohol according to a previous method with some modification^55^. During the extraction, the samples were kept in darkness and mixed many times until all the tissues became white. Finally, each sample was diluted tenfold with 96% alcohol, and the optical density was determined at 665, 649, and 470 nm with an ultramicro spectrophotometer (IMPLEN GMBH). Ninety-six percent alcohol was used for the control. We repeated this experiment three times for quantitative analysis.

### Grafting analysis

Grafting was conducted as previously described with minor modification^47^. WT and *rf* at the five-leaf stage were cut into two parts at the top of the hypocotyl; the upper part was used as the scion, and the lower part was used as the rootstock. The whole graft was then wrapped firmly with a grafting clip to ensure good contact. After 15 days, the grafts survived, and the total number of ARs on different types of grafted hypocotyl was counted.

### Quantitative real-time RT-PCR (qRT-PCR) analysis

Cotyledons and leaves from tomato seedlings at different leaf stages (at the one-, two-, three-, and four-leaf stages) were used to analyze gene expression in WT and *rf*. Total RNA was extracted using the Promega RNA extraction kit, and cDNA was synthesized using HiScript II 1st Strand cDNA synthesis Kit (Vazyme) according to the manufacturer’s instructions. qRT-PCR was performed using a Stratagene Mx3005P (Agilent Technologies) with ChamQ Universal SYBR qPCR Master Mix (Vazyme). Each analysis was based on three biological replicates, and error bars represent the standard deviation from the mean of three biological replicates. Gene-specific primers are listed in Supplementary Table S2, and *ACTIN* was used as an internal control.

Relative transcriptional values were determined by the 2^−∆∆CT^ method. Actin was used as the internal reference.

### Physiological analysis

At the red ripening stage, some yield traits, including plant height, stem diameter, weight per fruit, and fruit shape index, were analyzed in WT and *rf*. The fruit shape index was calculated by the maximum length divided by the maximum width. All corresponding fruits were used for evaluating weight per fruit. At least 50 random fruits for each line were included, and error bars represent the standard deviation of the biological replicates.

Plant height was measured from the soil surface to the plant terminal bud; the internode diameter and total fruit numbers per plant were also determined for the corresponding plants. The internode diameter at the middle position of each internode was measured using a Vernier caliper (with 0.01 mm resolution). The total fruit per plant was counted including all mature and young fruits. Quantification of each line included at least seven plants, and error bars represent the standard deviation of the biological replicates.

To quantify leaf area, we measured the terminal leaflet area of the third compound leaf using a scanner (Epson Perfection 4990 PHOTO). For each measured group, we used 12 tomato plants with similar growth conditions. Error bars represent the standard deviation of the biological replicates.

### **Accession numbers**

The genes used in this article can be found in the Solanum Genomics Network (https://solgenomics.net) under the following accession numbers: *AU* (Solyc01 g008930) and *SlHEMA1* (Solyc04 g076870).

## Supplementary information

Legends of supplemental Figure1-7
