# Supplementary material for: AUREA maintains the balance between chlorophyll synthesis and adventitious root formation in tomato
Source: Hortic Res. 2020 Oct 1;7:166. doi: 10.1038/s41438-020-00386-x (PMC7527990; doi:10.1038/s41438-020-00386-x)
Supplement: Supplementary file 1 — Legends of supplemental Figure1-7 [file 41438_2020_386_MOESM1_ESM.docx]

**Supplemental Data**

The supplemental materials are available.

**SUPPLEMENTAL FIGURE LEGENDS**

**Supplemental Figure S1.** Y-WT is susceptible to flooding.

**a-b**, Comparison of flooding resistance between WT and Y-WT at 0, 1, and 3 days post flooding (DPF). Bars=1 cm.

WT, wild type; Y-WT, yellow WT which was artificially created by darkness treatment.

**Supplemental Figure S2.** Fine identification of candidate gene

a, The segregation of AR phenotype and χ2 goodness-of-fit test ratios of segregation in F_2_ population.

b, The whole SNP loci in tomato chromosome 01; there were 402 SNPs were identified in chromosome 01, only two genes (red circle) were specially located at the peak with 1.0 (100%) SNP-index. Black dots represented SNP loci.

c, Functional annotation of those two SNP loci with 100% SNP-index in b.

**Supplemental Figure S3.** Expression level of *AU* in WT and *OE-AU/rf* plants.

*OE-AU/rf* represents overexpression of *AU* in *rf*. T1-47, 56 and 93 represent 3 independent lines of *OE-AU/rf* of T1 generation from different T0 plants*.* Error bar represents SE (n=3).

**Supplemental Figure S4. Temporal correlation of chlorophyll content and AR numbers**

a, Chlorophyll content at different stages of WT, *rf*, and *OE-AU/rf* plants. T1-47, 56 and 93 represent 3 independent lines of *OE-AU/rf* of T1 generation from different T0 plants*.* 1st–5th represents corresponding leaf stage. Error bar represents SE (n=3).

b, The number of ARs in *rf* at different leaf stage. 1st–5th represents corresponding leaf stage. Error bar represents SD (n=42, 42, 17, 7 and 7, respectively).

**Supplemental Figure S5. *AU* play dual function in stem expansion and leaflet area**

a, Diameter of the first four internodes of WT, *rf*, and *OE-AU/rf* plants.

T1-47, 56 and 93 represent 3 independent lines of *OE-AU/rf* of T1 generation. Error bar represents SE (n=19).

b, Terminal leaflet area in the third compound leaf of WT, *rf*, and transgenic plants. Error bar represents SE (n=12).

c, A schematic diagram indicates the terminal leaflet measured in b. The red dashed circle indicates the terminal leaflet measured in b.

**Supplemental Figure S6.** Differential recovery between WT and *rf* from flooding treatment.

**a**, WT and *rf* after 3 day flooding treatment. Both WT (uppanels) and *rf* (bottom panels). Bars=1 cm.

**b**, WT and *rf* after 5 day flooding treatment. The *rf* (bottom panels) exhibited higher resistance to flooding than WT (up panels). Bars=1 cm.

**Supplemental Figure S7.** Physiological phenotypes of WT, *rf* and transgenic plants.

**a**, Fresh weight per fruit in WT and *rf*. Error bar represents SE (n=50)*.*

The *rf*-6, 7, 10 represent three genetically stable lines derived from M1 generation.

**b***,* Plant height in WT, *rf* and *OE-AU/rf* plants. Error bar represents SE (n=7)*.*

**c**, Fruit shape index in WT and *rf.* The red mature fruits were used to determine the maximal length and width for the evaluation. Fruit shape index (FSI) was generated by maximal length divided by maximal width. The *rf*-6, 7, 10 represent three genetically stable lines derived from M1 generation. Error bar represents SE (n=50).

**d**, The total fruit numbers per plant in WT and *rf*. Error bar represents SE (n=7)*.*

**Supplemental Figures**

**
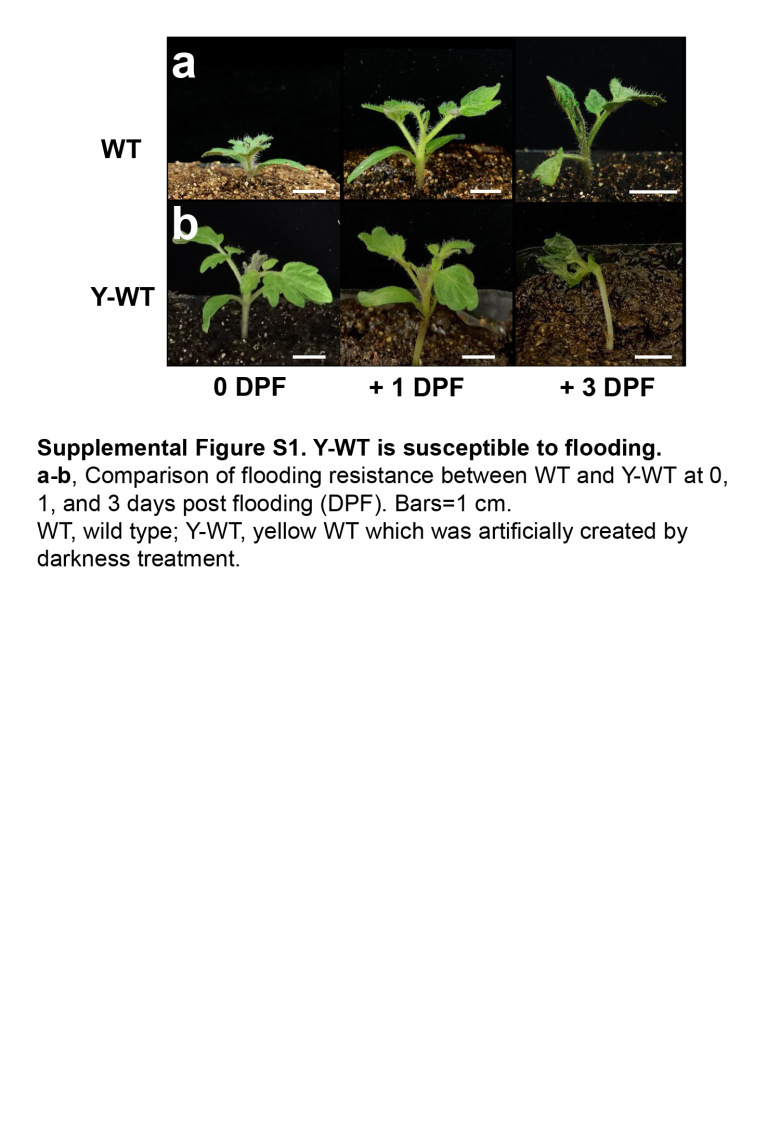
**

**
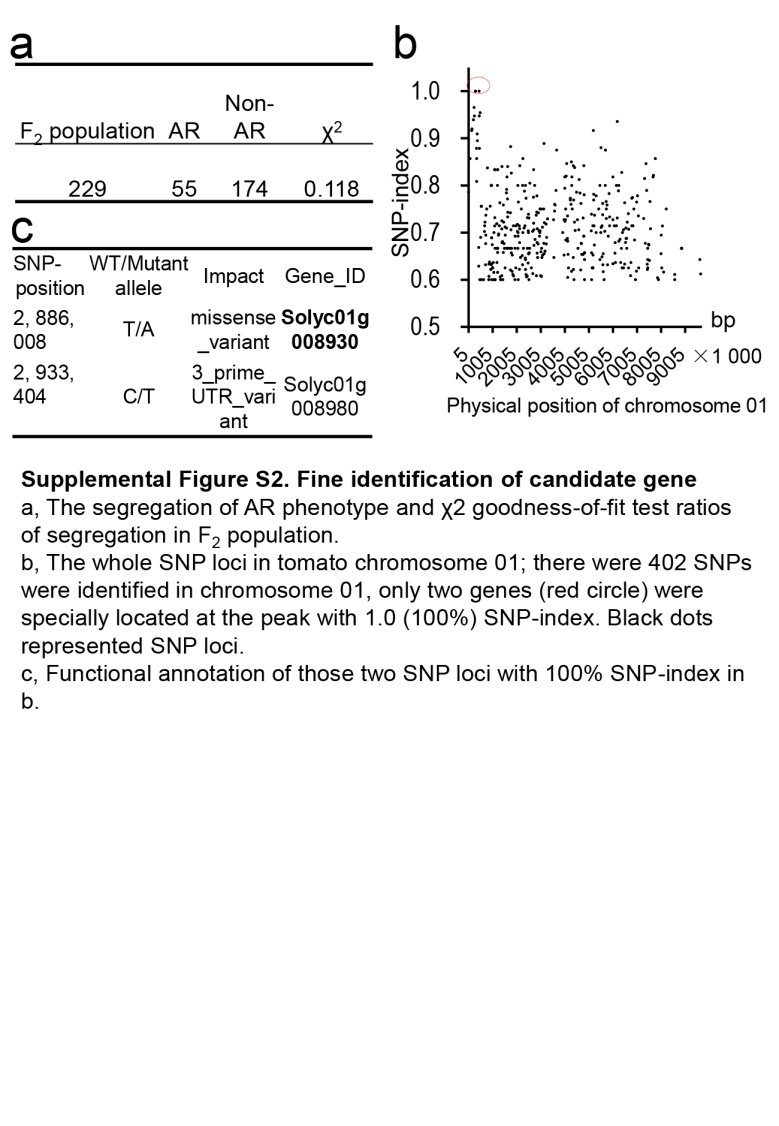
**


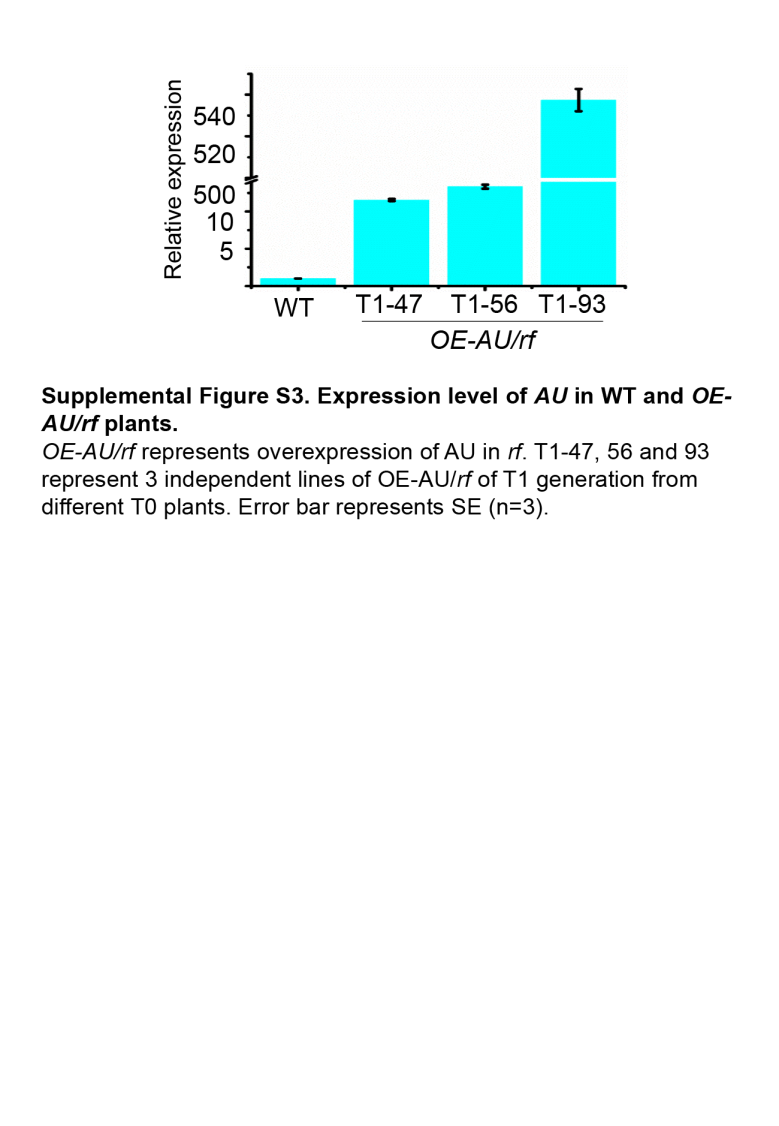


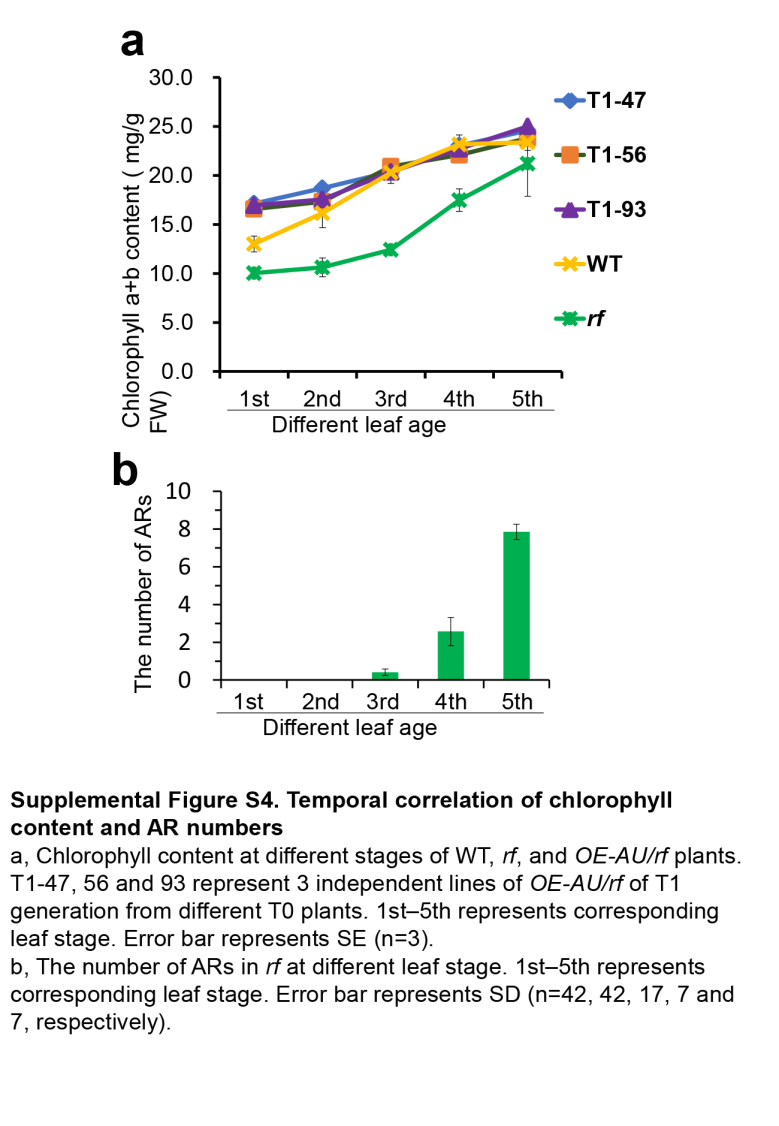


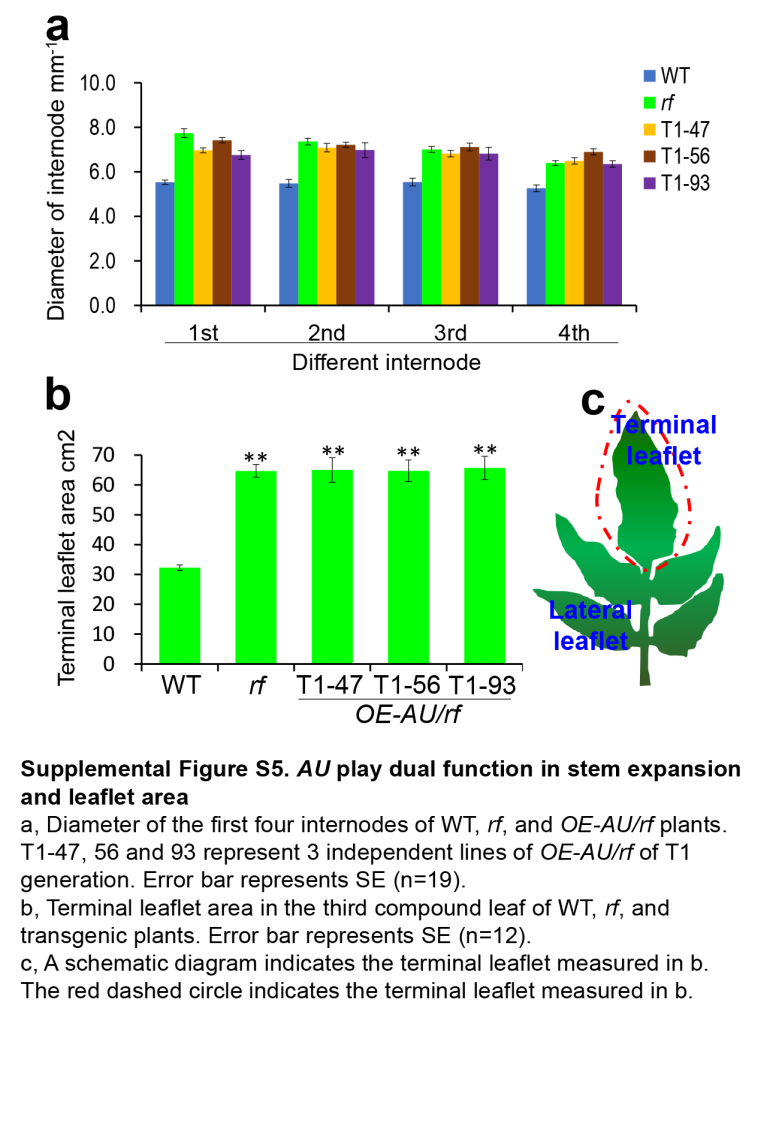


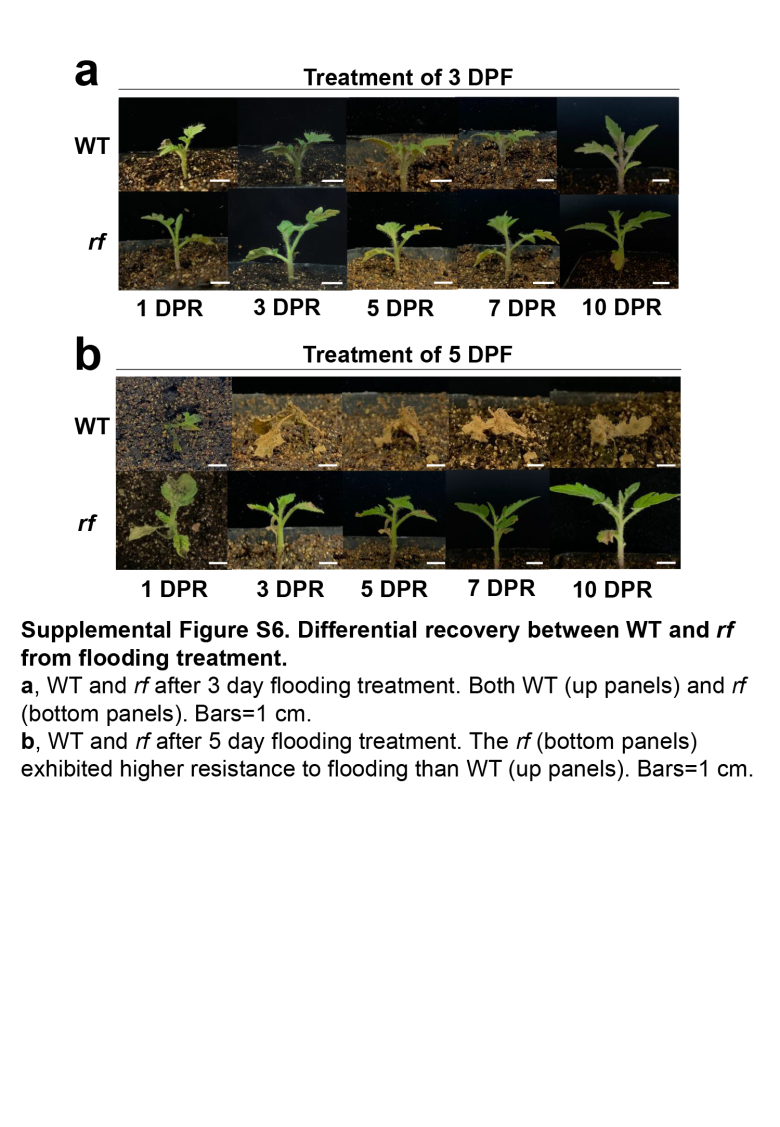


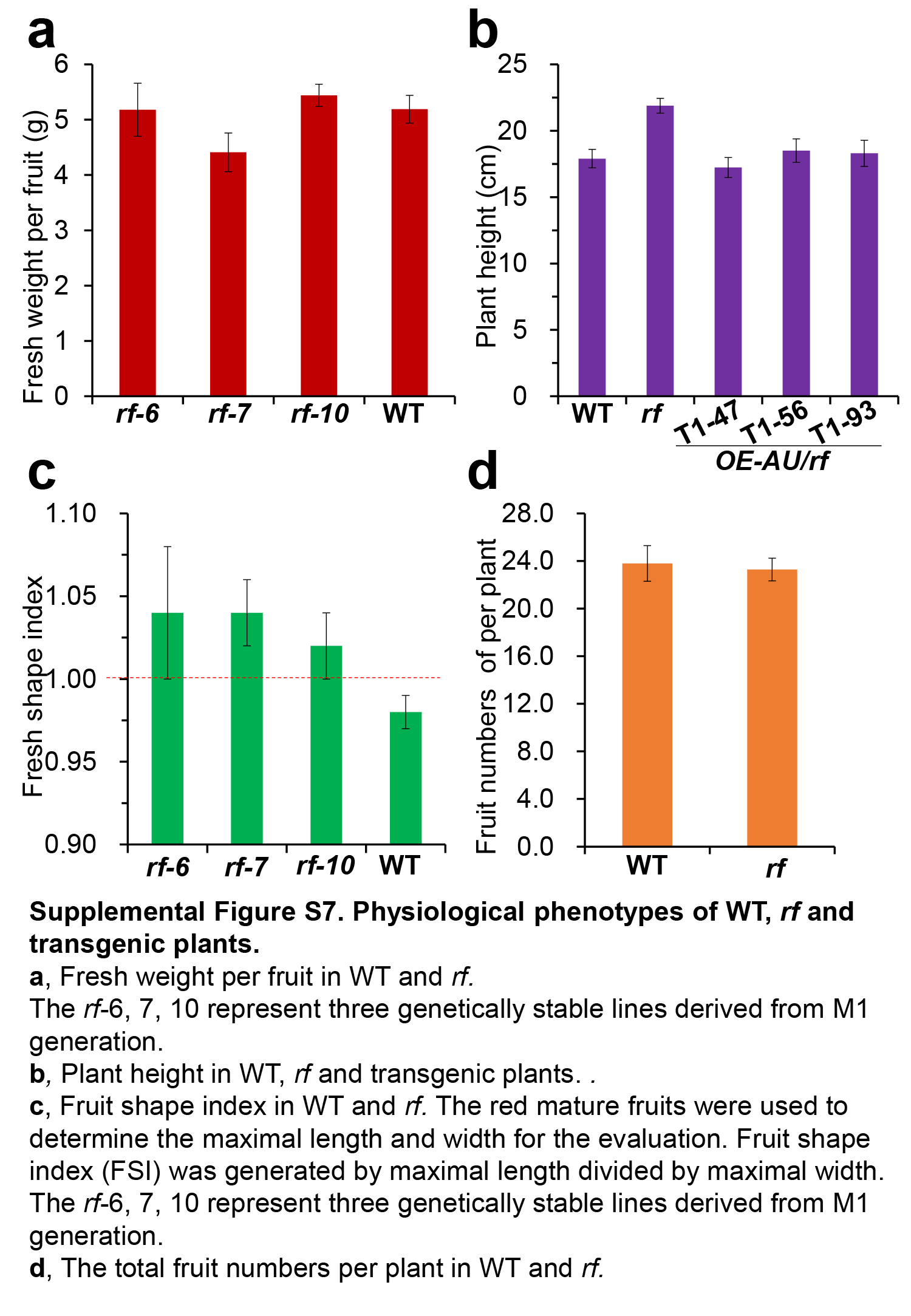


**Supplemental Tables**

**Supplemental Table S1.** The primers used in this work.

| Primers name | sequence (5' - 3') | Application |
| --- | --- | --- |
| 221-*AU*-F | ggggacaagtttgtacaaaaaagcaggctccatgGAGTGTTTTTCTTCACTAGGG | Cloning |
| 221-*AU*-R | ggggacaagtttgtacaagaaagctgggtcCATAGGCGATAGAGTTGAGATTGTC | Cloning |
| *SlHEMA1*-F | TCTTCTTCTAGCCTTTCTGCTCTTG | qRT-PCR |
| *SlHEMA1*-R | TCAGGAATAGCCAGTTTTTCACG | qRT-PCR |
| *ACTIN*-F | GTCCTCTTCCAGCCATCCA | qRT-PCR |
| *ACTIN*-R | ACCACTGAGCACAATGTTACCG | qRT-PCR |

Note, all capital letters indicate the gene primers, the small letters in cloning primers of *AU* represent the homologous recombination sequence.
